# The advent of structural biology *in situ* by single particle cryo-electron tomography

**DOI:** 10.1007/s41048-017-0040-0

**Published:** 2017-05-29

**Authors:** Jesús G. Galaz-Montoya, Steven J. Ludtke

**Affiliations:** National Center for Macromolecular Imaging, Verna and Marrs McLean Department of Biochemistry and Molecular Biology, Baylor College of Medicine, Houston, TX 77030 USA

**Keywords:** Cryo-electron tomography, Single particle tomography, Subtomogram averaging, Direct detection device, Contrast transfer function

## Abstract

Single particle tomography (SPT), also known as subtomogram averaging, is a powerful technique uniquely poised to address questions in structural biology that are not amenable to more traditional approaches like X-ray crystallography, nuclear magnetic resonance, and conventional cryoEM single particle analysis. Owing to its potential for *in situ* structural biology at subnanometer resolution, SPT has been gaining enormous momentum in the last five years and is becoming a prominent, widely used technique. This method can be applied to unambiguously determine the structures of macromolecular complexes that exhibit compositional and conformational heterogeneity, both *in vitro* and *in situ*. Here we review the development of SPT, highlighting its applications and identifying areas of ongoing development.

## INTRODUCTION: THE NEED FOR SINGLE PARTICLE TOMOGRAPHY

Over the last five years, thanks to the development of direct detection devices (DDDs) (Milazzo *et al.* 2005), cryo-electron microscopy (cryoEM) single particle analysis (SPA) has transitioned from being an established, but limited, technique to being at the forefront of structural biology (Eisenstein 2016; Nogales 2016). SPA can now achieve resolutions comparable to those of typical X-ray crystal structures while maintaining the specimen in a solution-like environment, thereby avoiding dehydration and crystallization artifacts. While a very powerful technique, SPA still suffers from two primary limitations: first, it is sometimes unable to unambiguously resolve reliable structures of macromolecules exhibiting continuous conformational flexibility; second, it cannot be directly used to study macromolecules within cells or other unique structures *in situ*.

Single particle tomography (SPT), also known as subtomogram averaging (STA), offers a solution to both of these problems. Indeed, with per particle 3D data, it is easier to unambiguously discriminate between changes in particle orientation versus changes in particle conformation, addressing the first issue above. Furthermore, the most impactful application of SPT lies in the cellular milieu. Since tomograms are a 3D representation of the imaged specimen, with SPT it is possible to isolate individual macromolecules from a cellular tomogram. These individual “subtomograms” can then be subjected to SPA-like 3D alignment and averaging, making true *in situ* structural biology at nanometer resolution feasible.

**Fig. 1:**
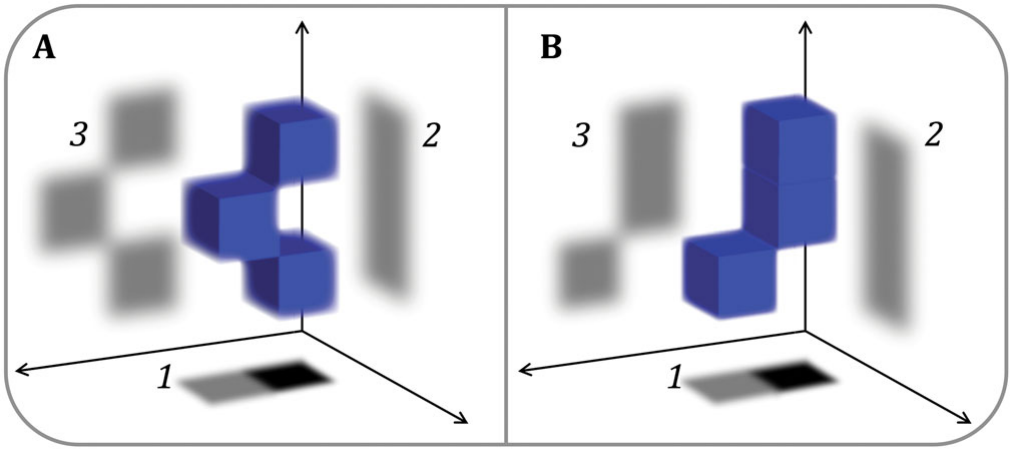
2D versus 3D imaging for particle classification. A simple conceptual demonstration that conformationally different particles (**A** and **B**) can yield multiple projections that are extremely similar (even if orthogonal), as shown by projections *1* and *2*, particularly given the high levels of noise typical of cryoEM micrographs of either *in vitro* or *in situ* specimens. To push structures to high resolution, it is critical to distinguish between projections of structurally different molecules that might otherwise be erroneously classified together and aligned based on overall low-resolution feature similarity. Collecting more than two images, as done in tomography, possibly from a different axis (as shown by projection *3* in the figure), can help to distinguish conformational differences among particles

The difficulty faced by SPA when studying particles undergoing large-scale continuous conformational change is the ambiguity produced by making projections of 3D objects. As conceptually outlined in Fig. 1, extremely different 3D structures can theoretically yield one or more indistinguishable projections, particularly given the high noise levels present in typical CryoEM images, both *in vitro* and *in situ*. With continuous conformational variability, it can thus be mathematically impossible to unambiguously distinguish changes in particle orientation from changes in particle conformation with only a single 2D image for each particle. These limiting factors, namely high levels of noise and conformational variability, are exacerbated for macromolecules *in situ*.

For macromolecules *in vitro*, the “tilt validation” method (Henderson *et al.* 2011, 2012) partially addresses the issue of confounding orientation and conformation by imaging isolated particles from two different directions to assess the reliability of orientation assignment in SPA reconstructions. However, this is a validation method and not a tool for initial analysis. The random conical tilt (RCT) (Radermacher *et al.* 1986) and orthogonal tilt reconstruction (OTR) (Leschziner and Nogales 2006) methods make use of this concept to reconstruct challenging structures, but have a number of other limitations. Furthermore, all these methods image the specimen from only two angles about the same axis and, again, are only applicable to isolated complexes.

In addition to allowing for the computational isolation of macromolecular complexes from cells, single particle tomography (SPT; also known as subtomogram averaging, or STA), can be viewed as an extension of the tilting concept of RTC and OTR by collecting multiple tilted views of single particles. This is literally “tomography of single particles,” as the inherent goal is to produce a tomographic 3D view for each individual particle in a system, be it *in vitro* or *in situ*. The particles are then processed through a pipeline akin to that of SPA cryoEM. That is, the tomographic single particles are (in simplified terms) aligned, classified by composition and/or conformation, and averaged as part of a standard pipeline that is applicable to both particles *in vitro* and *in situ*.

**Fig. 2:**
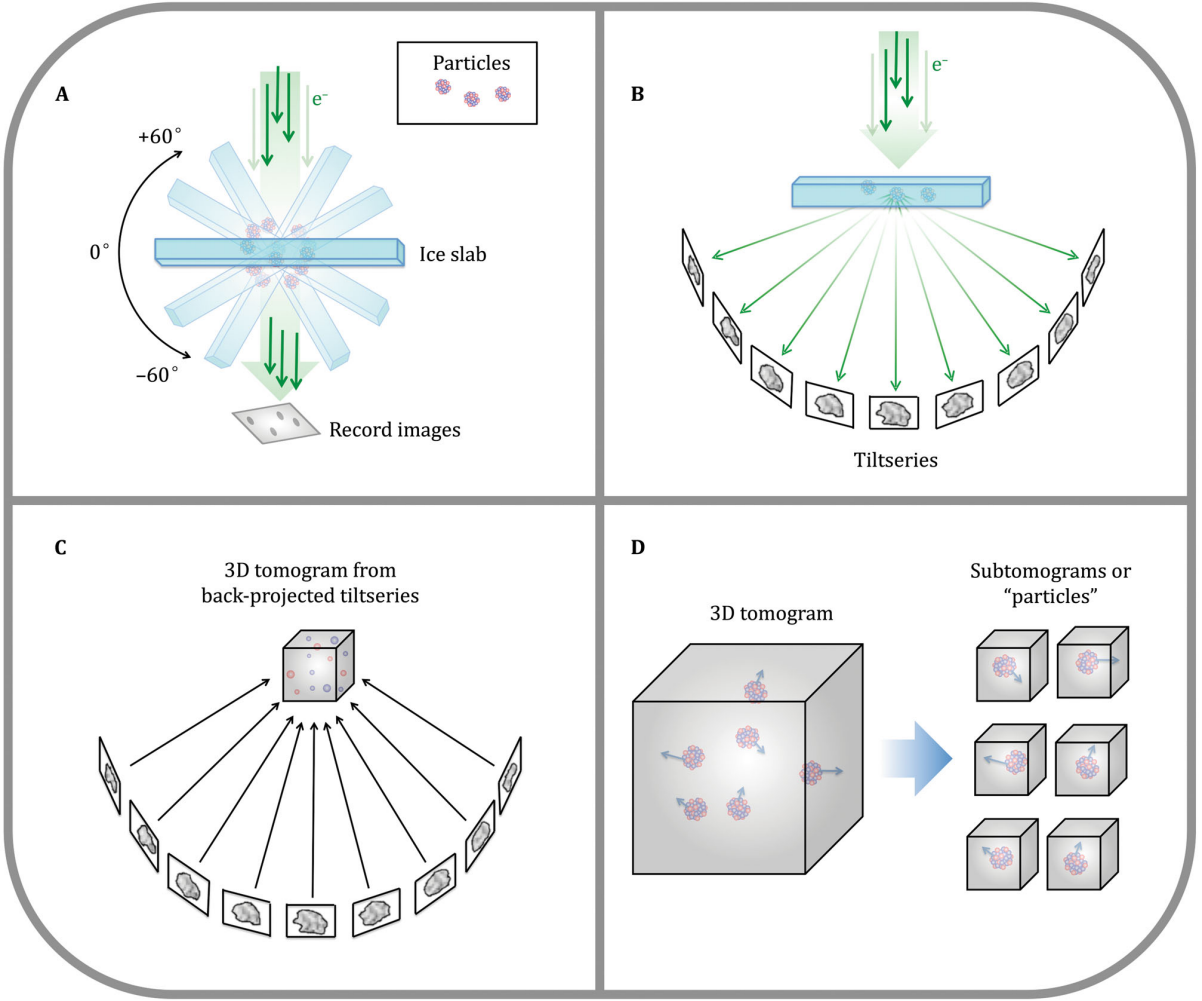
CryoET schematic. **A** In cryoET, the ice-embedded specimen, typically shaped as a slab, is tilted through a wide range of angles in the electron microscope and an image is recorded at each angle. **B** This collection of images around a common axis constitutes a “tiltseries.” **C** The images in a tiltseries can be computationally aligned to their common axis and reconstructed into a 3D tomogram by weighted back-projection or other methods. **D** Subtomograms representing a 3D view of individual macromolecules can be extracted from the reconstructed tomogram, then aligned and averaged (Fig. 7). (Partially inspired by Grünewald *et al.* 2002)

Although SPT has opened the window to increasing the resolution of structural biology *in situ* by averaging repeating features in cellular tomograms, this method also suffers from significant limitations. SPT builds on cryo-electron tomography (cryoET), which historically has been considered a low-resolution technique. In cryoET, a set of images (*i.e.*, a “tiltseries”) is collected for each specimen area by tilting the specimen stage through a range of angles, usually about a single axis (*e.g.*, ±60° in increments of 1°–5° or more). Very high cumulative electron dose (~50–120 e/A^2^) has been the norm to obtain sufficient contrast in each image of a tiltseries to permit accurate alignment, with the side effect of destroying high-resolution information progressively through the series. Each tiltseries can then be computationally reconstructed into a 3D tomogram representing the 3D structure of the imaged area (Fig. 2). The resolution of raw tomograms is highly anisotropic and remains somewhat ill-defined (Cardone *et al.* 2005), but is generally estimated to range from roughly 50–150 Å (depending on the specimen and on data collection parameters). While this is sufficient to resolve cellular organelles and identify large macromolecular complexes, it is not sufficient to resolve macromolecular structure in detail. Nonetheless, each individual subtomogram does contain some high-resolution information, which, upon averaging with other ostensibly identical particles, can be recovered to yield structures at much higher resolution, depending on imaging conditions.

**Fig. 3:**
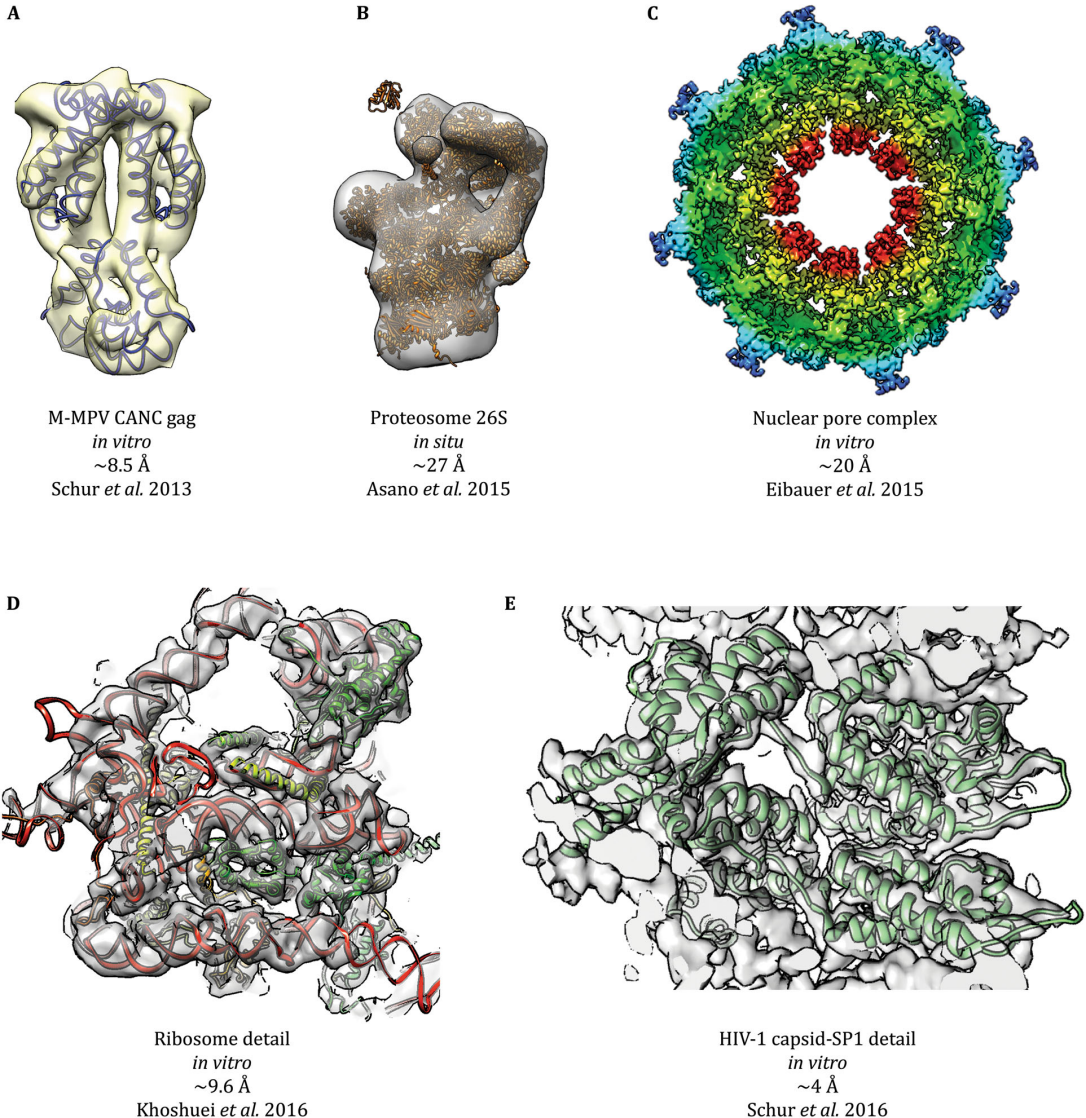
Sampling of notorious cryoSPT studies. This figure is a sampling of cryoSPT structures published at different resolutions, prepared directly from the corresponding deposited (emdatabank.org) maps and models. **A** The structure of M-MPV CANC Gag (EMD-2488) was the first one solved to subnanometer resolution by cryoSPT. **B** Asano *et al.* undertook the study of proteasomes inside intact neurons, resolving multiple states for the 26S proteasome (EMD-2830 is shown), making use of a Volta phase plate (VPP). **C** Nuclear pore complexes (NPCs) are some of the most challenging specimens studied by cryoSPT due to their large size, extreme conformational flexibility, and the need for a lipid environment. Eibauer *et al.* solved the structure of the *X. laevis* NPC at unprecedented resolution for this specimen (EMD-3005). A comparable resolution was recently achieved for another nuclear pore complex (not shown; Kosinski *et al.* 2016). **D** A recent proof-of-concept study demonstrated that the VPP could be used in cryoSPT experiments to solve the structure of particles without any symmetry, such as the ribosome, to subnanometer resolution, using a relatively small number of particles (*N* = 1400; EMD-3418). **E** The highest-resolution structure by cryoSPT to date is that of the immature HIV-1 CA-SP1 lattice (EMD-4015), which allowed building an atomic model (PDB-5L93)

The recent development of direct electron detectors, phase plate technology, and improved contrast transfer function (CTF) correction methodologies for cryoET have made it possible to achieve images with higher contrast and resolution, respectively, using much lower dose. Additionally, when averaging subtomograms, fewer images may be collected for each tiltseries, permitting the use of higher dose per image without impacting total dose. Indeed, these improvements have led to several recent cryoSPT studies approaching (Cassidy *et al.* 2015; Galaz-Montoya *et al.* 2016a; Khoshouei *et al.* 2016; Kudryashev *et al.* 2016; Li *et al.* 2016) or achieving (Bharat *et al.* 2015; Pfeffer *et al.* 2015a, b; Schur *et al.* 2013, 2015a, b; Mattei *et al.* 2016) subnanometer resolution, with the highest-resolution structure being solved to ~4 Å (Schur *et al.* 2016). Simplistic CTF correction (not accounting for defocus gradients) of cellular data has also demonstrated measurable improvements through SPT of microtubules *in situ* (Grange *et al.* 2017). Some of the most impressive results in cryoSPT during the last few years are shown in Fig. 3. Perhaps the greatest remaining limiting factor for *in situ* experiments is specimen thickness, which limits electron penetration, making it impractical to study specimens thicker than roughly 0.5–1.0 μm. The study of thicker eukaryotic cells requires significant physical manipulation, such as slicing the specimen into thin sections (Al-Amoudi *et al.* 2004).

Continued development of computer technology has also played a critical role in improving the resolution achievable by cryoSPT. A single cellular tomogram (including those with repeating features that can be averaged) can exceed 64 GB in size at full resolution; therefore, the reconstruction of hundreds of tomograms is extremely computationally intensive, particularly if iterative reconstruction methods are used without downsampling. Per-particle computational costs (*i.e.*, preprocessing, alignment, classification, and averaging of subtomograms) are several orders of magnitude greater for SPT than for SPA. However, Moore’s Law (Schaller 1997) has finally caught up with this field, and it is now practical to compute hundreds of tomographic reconstructions and average tens of thousands of subtomograms making use of algorithms that previously would have been untenable (Agulleiro *et al.* 2012).

## HISTORY AND APPLICATIONS OF SINGLE PARTICLE CRYO-ELECTRON TOMOGRAPHY

The mathematical foundations underlying 3D image reconstruction date to 1917 when Johann Radon demonstrated that a function could be precisely reconstructed from an infinite set of its projections (Hawkes 2007). Since then, many mathematical techniques have been devised to reconstruct a 3D model from a set of 2D projection images, for medical imaging and a wide range of other applications, including transmission electron microscopy (TEM) tomography.

The theory underlying electron tomography (ET) and its application to study biological specimens (De Rosier and Klug 1968a, b; Hart 1968; Hoppe *et al.* 1968), including individual metal-stained macromolecules (Hoppe *et al.* 1974) and averages of a few subvolumes (Knauer *et al.* 1983; Oettl *et al.* 1983), were first demonstrated decades ago. However, there were many experimental and computational barriers to widespread adoption of ET at that time.

**Fig. 4:**
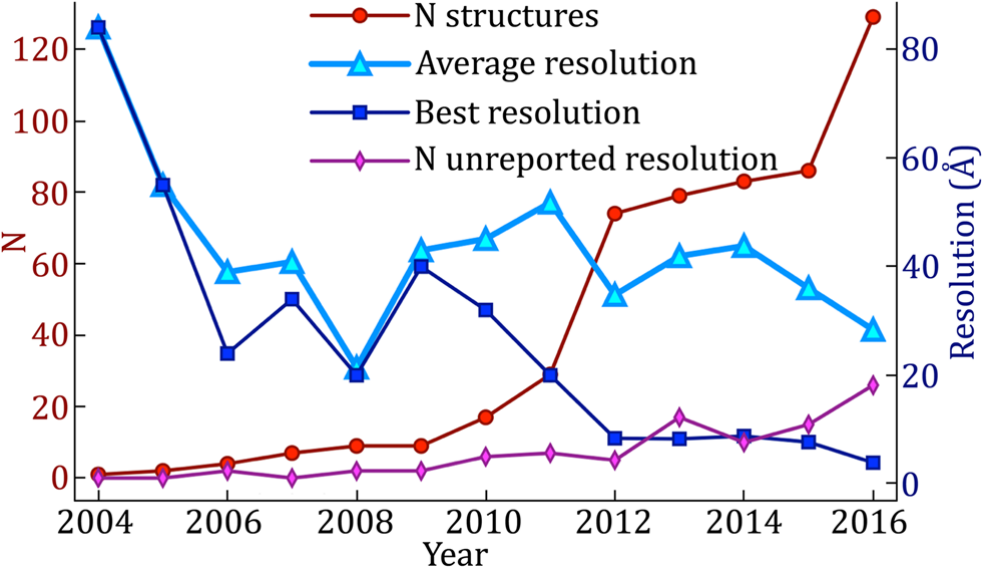
Increase in yearly structures deposited in the EMDB by SPT from 2004 to 2016 and resolution improvement trend. While the highest resolution achieved each year has continued to improve, the average resolution is improving only very gradually

The development of cryo-electron microscopy (cryoEM) was a major breakthrough that demonstrated that biological specimens, including cells and “single particles,” could be better preserved in vitrified water solutions, free from crystallization and staining artifacts (Dubochet *et al.* 1981, 1988), in a close-to-native state. CryoEM was first applied to 2D protein crystals (Taylor and Glaeser 1974) and was demonstrated for isolated particles (viruses) a decade later (Adrian *et al.* 1984). It took many more years before Walz *et al.* completed the first cryoSPT experiments in 1997, in their study of thermosomes *in vitro*. They showed that the structure of this chaperonin could be determined without missing wedge artifacts by computing tomograms of the specimen in solution, extracting volumes (*i.e.,* subtomograms) containing individual thermosomes, and averaging them after correct alignment. Since then, the publication rate of studies using SPT has been accelerating. Figure 4 shows the number of yearly structures solved by SPT and deposited in the Electron Microscopy Data Bank (EMDB) from 2004 to 2016, as well as the best resolution achieved in each of those years. A few structures that were not deposited to the EMDB were solved in 1997, 1998, and 2003, as noted in previous reviews (Schmid 2011; Kudryashev *et al.* 2012). The resolution averages presented in Fig. 4 exclude structures for which no resolution was reported (pink line). Of note, averages of at least two particles should always report an estimate of the resolution, however modest it might be. This will facilitate the interpretation of features in the structures being published and in the figures being displayed.

Early SPT relied largely on undocumented *ad hoc* scripts (Winkler and Taylor 1999; Pascual-Montano *et al.* 2002; Förster *et al.* 2005; Nicastro *et al.* 2006; Schmid *et al.* 2006; Winkler 2007; Schmid and Booth 2008). However, the methodology has gradually become more accessible with the surge of open-access software for SPT, such as AV3 (Förster and Hegerl 2007), PEET (Nicastro *et al.* 2006) (available through IMOD (Kremer *et al.* 1996)), Jsubtomo (Huiskonen *et al.* 2010), PyTom (Hrabe *et al.* 2012), Dynamo (Castaño-Díez *et al.* 2012), EMAN2 (Galaz *et al.* 2012; Murray *et al.* 2014; Galaz-Montoya *et al.* 2015), and RELION (Bharat *et al.* 2015).

The evolution of cryoET can be followed through reviews in this discipline over the last couple of decades, (*e.g.*, Koster *et al.* 1997; Baumeister 2002; Förster *et al.* 2005; Crowther 2010; Fernández 2012). CryoET can now be routinely applied to study macromolecules in solution (Medalia *et al.* 2002a) and in their native cellular context (Medalia *et al.* 2002b; Ortiz *et al.* 2006, 2010; Brandt *et al.* 2010; Schwartz *et al.* 2012). Challenging specimens whose structures have been solved by cryoSPT include complexes that exhibit extensive structural heterogeneity, such as carboxysomes (Schmid *et al.* 2006), dynein interacting with microtubules along axonemes (Nicastro *et al.* 2006), and pleomorphic viruses (Harris *et al.* 2006, 2013; Huiskonen *et al.* 2010; Schmid *et al.* 2012). CryoSPT has also been used to study viruses infecting their host cells (Hu *et al.* 2013; Peralta *et al.* 2013; Sun *et al.* 2014; Riedel *et al.* 2017; Murata *et al.* 2017), even in transient conformations along their assembly pathway inside cells (Dai *et al.* 2013). Much smaller complexes, such as the proteasome in different conformational states, have also been visualized *in situ*, inside neurons (Asano *et al.* 2015) (Fig. 3). Other complex systems whose structures have been best characterized using SPT are flagellae (Koyfman *et al.* 2011; Carbajal-González *et al.* 2013; Zhao *et al.* 2013), polysomes *in situ* (Brandt *et al.* 2010), membrane-bound ribosomes (Pfeffer *et al.* 2012, 2015a, b), nuclear pore complexes (Stoffler *et al.* 2003; Maimon *et al.* 2012; Eibauer *et al.* 2015; Kosinski *et al.* 2016), and other membrane-bound complexes (Davies *et al.* 2011; Eibauer *et al.* 2012; Dalm *et al*. 2015; Nans *et al.* 2015; Briegel *et al.* 2016; Sharp *et al.* 2016), as well as amyloid protein aggregates interacting with chaperones (Shahmoradian *et al.* 2013; Darrow *et al.* 2015), among others. A recent review (Asano *et al.* 2016) and a book chapter (Wan and Briggs 2016) describe in detail the technical aspects of carrying out SPT analyses.

## CHALLENGES IN SINGLE PARTICLE TOMOGRAPHY

While cryoSPT is now being successfully applied to many biological problems that could not be addressed in near-native conditions a few years ago, it is also important to understand its current limitations and their underlying sources.

### Radiation damage

A fundamental limitation in any cryoEM/ET study is the unavoidable fact that the specimen is being destroyed as it is being imaged. Thus, the permissible dose is limited to preserve detailed features (Glaeser 1971; Grubb 1974). This problem is exacerbated in tomography, since many tilt images of the same specimen area must be collected, and yet each tilt image must contain sufficient information for accurate tiltseries alignment to yield a high-fidelity reconstruction. How to optimally allocate the total cumulative dose among all the images of a tiltseries depending on the particular goals of a study (*i.e*., “dose fractionation”) has been a longstanding problem in cryoET (McEwen *et al.* 1995) and is still being actively researched (Hagen *et al.* 2017). A recent clever technique to turn the radiation sensitivity of biological specimens into an advantage is the concept of “bubblegrams” (Cheng *et al.* 2012; Wu *et al.* 2012) and “tomo-bubblegrams” (Fontana *et al.* 2015), in which the varying radiation sensitivity of different molecular species can be used to localize and identify substructures, while still preserving high-resolution detail in the early portion of the exposure. Aside from such unorthodox tricks, however, radiation damage remains the primary limiting factor in cryoEM and cryoET (Cosslett 1978; Glaeser and Taylor 1978; Baker and Rubinstein 2010). Indeed, radiation damage is a complex phenomenon that remains under active investigation, since it depends not only on the chemical composition of the specimen but also on multiple data collection parameters such as cumulative dose, imaging temperature (Comolli and Downing 2005; Iancu *et al.* 2006; Bammes *et al.* 2010), and dose rate (Chen *et al.* 2008; Karuppasamy *et al.* 2011), among others. The recent development of DDDs has reduced the impact of radiation damage by permitting the recovery of a larger fraction of information at lower cumulative doses owing to the detector’s higher detective quantum efficiency (DQE) and improved modulation transfer function (MTF) (Milazzo *et al.* 2010).

### Dose fractionation, ice thickness, and beam-induced specimen motion

**Fig. 5:**
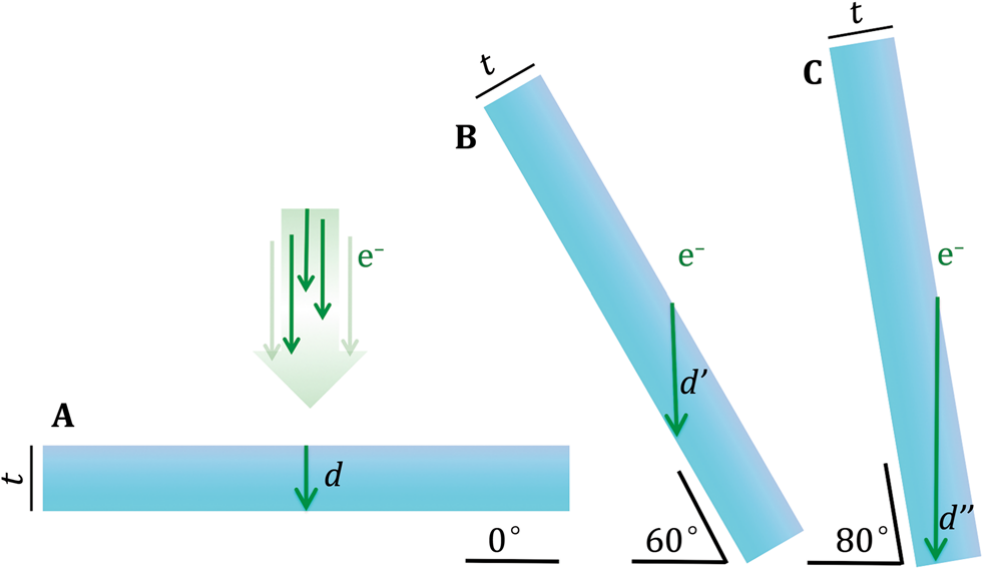
The effective ice thickness of slab-shaped specimens increases with tilt angle. Even for relatively thin specimens (**A**), in cryoET the path of the electron beam through the slab-shaped specimen (*green arrows*, labeled *d*, *d’*, and *d’’*) increases dramatically with tilt angle (**B**, **C**). This limits the thickness *t* of cellular material that can be studied by cryoET and the maximum tilt angle at which usable data can be collected. Indeed, greater ice thickness degrades image quality and contrast as it increases charging, blurring, and multi-scattering events that cause images to be noisier

The SNR and contrast of an electron micrograph also depend on the thickness of the ice in which the specimen is embedded. In cryoET, the effective ice thickness scales with the secant of the tilt angle (Fig. 5). Images from tilt angles any higher than ±65° are often unusable and can decrease the quality of the tomographic reconstruction if included. The inability to collect a complete tomographic tiltseries (tilting through ±90°) causes the so-called “missing wedge” artifact. This term refers to the wedge-shaped region of Fourier space that is empty due to missing tilt images. This artifact produces anisotropic resolution in tomograms (Radermacher 1988) and poses one of the most critical problems for alignment and classification of subtomograms, as discussed further below.

When the specimen is too thick for beam penetration, it can be sectioned with a diamond knife (Al-Amoudi *et al.* 2004) or milled thin with a focused ion beam (FIB) (Marko *et al.* 2007) prior to imaging. While this enables the study of thicker specimens by cryoET, cryo-sectioning is, unfortunately, an extremely challenging technique that yields sections laden with artifacts, from compression and curving to crevasses and cracks (Al-Amoudi *et al.* 2003, 2005). On the other hand, while FIB milling produces less severe artifacts, it destroys the bulk of the specimen, and it can be very difficult to focus the milling process on the region of interest. An alternative method to study thick frozen-hydrated specimens is X-ray tomography (Wang *et al.* 2000; Le Gros *et al.* 2005), albeit typically at resolutions an order of magnitude lower than cryoET.

**Fig. 6:**
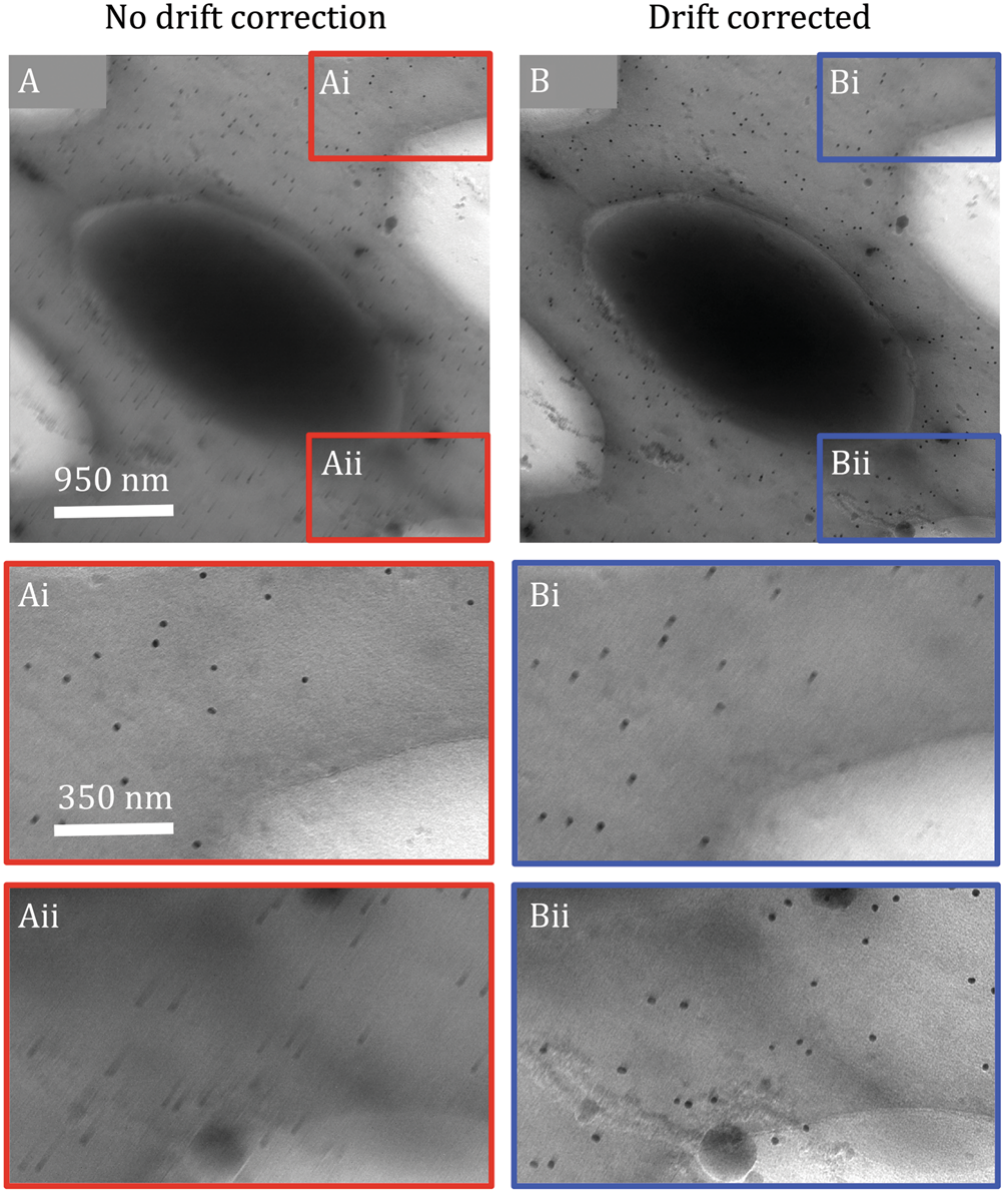
Incomplete, local unblurring of cryoET images by whole-frame motion correction. Image of a mouse platelet at 57° tilt without (**A**) and with (**B**) motion correction applied to 21 frames collected with a K2 DDD on a JEM3200FSC microscope. Blurring is anisotropic both before (**Ai** and **Aii**) and after (**Bi** and **Bii**) whole-frame motion correction. While motion correction by iterative frame alignment (Galaz-Montoya et al. 2016b) improves the overall image (**B**), the extent of improvement varies in different parts of the image (**Bii**). Unexpectedly, a region that was not originally blurry (**Ai**) becomes blurry after motion correction (**Bi**) while a different region is effectively unblurred (**Aii** versus **Bii**). This suggests that different parts of the specimen are subject to divergent apparent motions, possibly due to charging effects and/or motion perpendicular to the imaging plane, and therefore local motion correction methods are needed for cryoET images, similar to those applied per particle in SPA cryoEM

The conductivity of holey and/or continuous amorphous carbon support films commonly used in cryoET is decreased at cryogenic temperatures, resulting in the accumulation of charge during imaging. Charging and beam-induced motion are exacerbated by specimen curvature (*i.e.*, frozen menisci)(Chen *et al.* 2008) and ice thickness (Brink *et al.* 1998), and are therefore more prominent and more likely to occur in cryoET the higher the tilt angle (Galaz-Montoya *et al.* 2016a). Although DDDs permit collecting images as short movies, which can be corrected for beam-induced motion (Brilot *et al.* 2012), different particles in the imaged specimen may move in different directions (Campbell *et al.* 2012). Strategies have emerged to correct for this in SPA cryoEM, where individual particles can be motion corrected (Scheres 2014). However, extensive dose fractionation in cryoET with DDDs typically yields movie frames at each tilt angle with considerably lower contrast and higher noise than movie frames in SPA cryoEM. Aligning whole movie frames can produce micrographs that are only locally unblurred, sometimes blurring areas of the image that were not originally blurry. This can happen both in thick, cellular tomograms (Fig. 6), as well as in tomograms of isolated macromolecules (not shown). Single particles are not always readily detectable in the individual frames of movies in cryoET images. Furthermore, cryoET images often cannot be completely broken down into discrete particles because the specimen is continuous (*e.g.*, cells). Therefore, local unblurring strategies that can correct extremely low-dose images anisotropically and without discontinuities are needed to maximize productive data usage in cryoET, as recently proposed for SPA cryoEM (Zheng *et al.* 2016). Given the extensive dose fractionation and greater radiation damage due to total cumulative dose, accomplishing this for cryoET is foreseeably more challenging than for SPA cryoEM. To a first approximation, gold fiducials might serve the purpose of guiding local motion correction for extremely low-dose cryoET movie frames, in addition to serving their normal role in tiltseries alignment.

### Tiltseries alignment

Gold fiducials are commonly used as markers in cryoET so that the precise 3D orientation of each image in a tiltseries can be determined. Unfortunately, such gold markers have been shown to undergo independent beam-induced motions and therefore their positions are not strictly consistent and predictable across a tiltseries (Comolli and Downing 2005; Noble and Stagg 2015). This effect varies considerably among specimens and can be a limiting factor in reconstruction quality. Furthermore, gold fiducials cause extremely strong artifacts (“streaks”) that can obscure features in tomograms and interfere with subtomograms alignment. Strategies to regularize gold fiducial artifacts have been proposed (Song *et al.* 2012; Maiorca *et al.* 2014; Han *et al.* 2015), some of which are available in IMOD, but do not seem to be routinely applied. Alternatively, many fiducial-less alignment methods have been developed (Liu *et al.* 1995; Brandt *et al.* 2001; Renken and McEwen 2003; Castaño-Díez *et al.* 2007) but usually require that the specimen itself exhibits high-contrast features. These procedures are still prone to error since specimens can also undergo deformations with cumulative dose, and in most cases gold fiducials yield better tiltseries alignment. While subnanometer resolution can be achieved in cryoSPT with standard fiducial-based alignment (Schur *et al.* 2013), circumventing the known errors inherent in this methodology can improve resolution (Bartesaghi *et al.* 2012). Indeed, if made easily applicable, overcoming the limitations of fiducial-based alignment (Iwasaki *et al.* 2005; Bartesaghi *et al.* 2012; Zhang and Ren 2012) might facilitate achieving subtomogram averages beyond the 8–12 Å resolution range routinely for various types of specimens.

### Tomographic reconstruction methodologies

A century ago, Johann Radon postulated that a function could be precisely reconstructed from an infinite set of its projections (Radon 1917). This concept was a stepping-stone in the development of computed tomography (CT), which has found applications in medical imaging, astronomy, and TEM (Van Heel 1987). Indeed, a variety of methods have been developed to reconstruct the 3D structure of biological specimens from a set of 2D TEM projection images in known orientations. Mathematically, all of these methods are based on the central section theorem (DeRosier and Klug 1968a, b; Crowther *et al.* 1970; Crowther 1971), according to which the Fourier transform (FT) of a 2D projection from a 3D specimen corresponds to a section through the center of the specimen’s representation in Fourier space. Thus, one can construct the 3D FT of the specimen by combining the FTs of all the 2D projection images inserted into a 3D FT volume, and computing the inverse Fourier transform of the volume (*i.e.*, “direct Fourier inversion”) (Ludtke *et al.* 1999; Belnap 2015). Although this approach is conceptually simple, interpolation in Fourier space is non-trivial, and different strategies produce different artifacts. While Fourier inversion has become the standard approach in SPA (Penczek *et al.* 2004; Penczek 2010), it is not widely used in cryoET (Heymann and Belnap 2007).

The most popular reconstruction method for cryoET due to its speed and relatively easy implementation for large volumes is weighted back-projection (WBP), a real-space equivalent of the central section theorem (Gilbert 1972; Radermacher 2007). It consists of literally “projecting back” the densities of 2D projections as rays into a 3D reconstruction volume. Appropriate weighting of the back-projected densities is needed to avoid the implicit low-pass filtering effect of WBP. However, WBP yields reconstructions with very poor contrast and strong “streaking” artifacts compared to iterative algebraic reconstruction methods such as the simultaneous iterative reconstruction technique (SIRT) (Gilbert 1972) or algebraic reconstruction techniques (ART) (Marabini *et al.* 1998). On the other hand, iterative algebraic methods are typically much slower, can diverge for some datasets or sometimes destroy high-resolution information, and determining *a priori* the optimal number of iterations to use and other parameters is not generally possible.

The problem of tomographic reconstruction is still being actively researched, with many novel methods described over the last decade (Díez *et al.* 2007; Batenburg and Sijbers 2009; Wan *et al.* 2011; Kunz and Frangakis 2014; Turoňová *et al.* 2015; Zhou *et al.* 2015; Chen *et al.* 2016). A few methods have been proposed to recover some of the missing information in limited-angle tomography, for example, by using convex projections (Carazo and Carrascosa 1987). Recently, the reconstruction method proposed by Chen and Förster (2014) was shown to restore some of the missing information by iterative extrapolation. Total variation minimization (TMV, or “regularization”) based on compressed sensing (CS) (Donoho 2006) has been applied successfully to ET in material sciences, yielding structures with minimal missing wedge artifacts and improved contrast (Saghi *et al.* 2011; Goris *et al.* 2012; Leary *et al.* 2013). Variations of this method have also been demonstrated for cryoET specimens (Aganj *et al.* 2007; Song *et al.* 2012). Recently, the iterative compressed-sensing optimized non-uniform (ICON) reconstruction (Deng *et al.* 2016) method demonstrated that CS can restore missing information in noisy cryoET data of both cells and isolated macromolecules, thereby minimizing missing wedge artifacts and yielding measurably better reconstructions than WBP.

At present, no single optimal algorithm has emerged, and the vast majority of users adopt whichever algorithm is most conveniently available or recommended by the software they have selected for tiltseries alignment. Reconstruction methods need to be carefully chosen depending on the goals of the study in question and the data collection parameters. For example, the success of TMV has been reported to depend on the tilt scheme used during data collection, while SIRT is less sensitive to variations in total dose or tilt scheme (Chen *et al.* 2014). It is important to note that certain algorithms are optimized for direct interpretation of tomograms but can destroy high-resolution information and are therefore suboptimal for averaging subtomograms if achieving higher resolution is the end goal. For example, SIRT delivers tomograms with much better contrast than WBP at the expense of introducing artifacts at high resolution. Furthermore, some algorithms rely on the individual images in a tiltseries having high contrast, such as the filtered iterative reconstruction technique (FIRT) (Chen *et al.* 2016), which does not seem to provide any advantages over WBP when applied to cryoET data. Interestingly, it has also been proposed that several reconstruction techniques can be applied sequentially to guide the choice of optimal parameters. For example, an initial SIRT reconstruction can guide the selection of the regularization parameter for TMV reconstruction, which can in turn help to choose adequate gray values to run a final reconstruction with the discrete algebraic reconstruction technique (DART) (Goris *et al.* 2013).

### Contrast transfer function determination and correction for tilted specimens

The contrast transfer function (CTF) (Erickson and Klug 1971) of the electron microscope modifies the amplitude of the signal in cryoEM micrographs (Toyoshima and Unwin 1988) in an oscillatory, resolution-dependent manner. While it is a function of several parameters, the only one that varies significantly during an imaging session is the defocus. In SPA cryoEM, CTF correction is a well-established, largely automated, and straightforward process, with various approaches achieving comparable results. Indeed, the “CTF challenge” recently compared many of the multiple algorithms available to perform CTF correction in SPA cryoEM (Marabini *et al.* 2015). On the other hand, as the defocus is directly related to the specimen height in the column with respect to the focal plane in the electron microscope, ET specimens produce a CTF that varies across the imaging plane due to the tilted geometry and, to a lesser extent, due to the thickness of the specimen. Compensating for these defocus gradients in images of cryoET tiltseries requires more complicated correction strategies than those implemented for SPA cryoEM. Without CTF correction, the resolution of subtomogram averages will typically be limited to 20**–**100 Å, depending on the imaging parameters. Resolving the structure of macromolecules by cryoSPT to better than 20 Å resolution is not yet a routine procedure, with the yearly average resolution being above this threshold (Fig. 4).

Several approaches have been implemented to determine the CTF and/or correct for it in cryoET data (Mindell and Grigorieff 2003; Winkler and Taylor 2003; Fernandez *et al.* 2006; Xiong *et al.* 2009; Zanetti *et al.* 2009; Voortman *et al.* 2011), with the most successful approach so far being that by (Schur *et al.* 2013). The latter approach achieved cryoSPT *in vitro* at subnanometer resolution for the first time, and has continued to yield structures at even higher resolution when combined with modern instrumentation, such as DDDs, and algorithmic improvements in image processing. The paramount achievement of Schur *et al.* 2013 using images taken with a charge-coupled device (CCD) was initially heavily dependent on stage eucentricity and stability during data collection and accurate autofocusing, as well as on a high particle density and automated data collection. Thin ice was also essential, as the specimen was assumed to be co-planar throughout the tomograms. So, while this was an effective proof of concept, these conditions might not be straightforward to achieve in a typical lab for all specimens. A year prior, a hybrid methodology combining concepts and data processing strategies from SPA cryoEM and cryoSPT resolved GroEL at subnanometer resolution as well (Bartesaghi *et al.* 2012), but this hybrid approach cannot be easily applied to cellular data. Another successful method (Eibauer *et al.* 2012) also recently achieved subnanometer resolution (Bharat *et al.* 2015), making use of a pair of additional high-contrast images collected away from the imaging area to interpolate the defocus in the region of interest. While this method was successful, it significantly increased data collection and processing complexity and assumed that the cryoEM support grid was flat (though not necessarily parallel to the imaging plane). Unfortunately, grid bending and “cryo-crinkling” of the carbon support mesh are common artifacts (Booy and Pawley 1993). Therefore, there is no guarantee that interpolation of the defocus at the imaging site by measuring the defocus in adjacent sites several microns away will always be accurate. Indeed, increased exposure near the imaging area due to lengthy focusing routines can induce deformations that compromise the accuracy of tiltseries alignment (Khoshouei *et al.* 2016).

Recently, a per-particle CTF correction method in 3D for cryoSPT was proposed (Galaz-Montoya *et al.* 2015) and demonstrated *in vitro* at near subnanometer resolution, without making any of the aforementioned assumptions (accurate defocusing during data collection, thin ice, unbent specimen, etc.), using only a few hundred icosahedrally symmetric virus particles (Galaz-Montoya *et al.* 2016a). Most importantly, the defocus gradient due to tilting was fitted by directly measuring the power spectrum in strips of constant defocus, similar to the method proposed by Fernández *et al.* (2006), except that it was done on each individual image (*i.e.*, different images in a tiltseries were never combined and the defocus gradient was linearly fit on a per-image basis, instead of relying on a single value from the central region of the image to compute the gradient). Indeed, DDDs now allow measuring the defocus directly from each image in a tiltseries, even at high tilt, which is essential under experimental settings for which the actual defocus might significantly differ from the target defocus and vary widely across the images of a tiltseries.

Performing CTF correction in 3D for tomographic reconstructions, including per-particle corrections (Bharat *et al.* 2015), has further been demonstrated to yield improvements compared to corrections considering 2D information only (Kunz and Frangakis 2016), as first theoretically proposed for virus reconstructions in SPA cryoEM nearly two decades ago when the depth of field started to become a resolution-limiting factor (Jensen and Kornberg 2000). The method proposed by Jensen and Kornberg to compensate for the depth of field was later generalized mathematically (Kazantsev *et al.* 2010).

### The missing wedge

The effective thickness of ET specimens increases with the secant of the tilt angle, meaning that at 60° the specimen is twice as thick than at 0°, and at 70° it is nearly three times as thick. This effect degrades image quality rapidly at higher tilt angles and, in most cases, ~60° is the highest tilt worth expending dose on. This means that typical ET tiltseries span only $\sim{2}/{3}$ of complete tomographic angular sampling. The missing angular range is termed the “missing wedge,” and leads to a variety of 3D reconstruction artifacts (Radermacher 1988), where features or particles are distorted in different ways, depending on their orientation with respect to the missing wedge.

Data collection methods alternative to canonical single-axis ET have been proposed to reduce the deleterious effects of the missing wedge in cryoET, such as dual-axis tomography (Penczek *et al.* 1995; Mastronarde 1997; Tong *et al.* 2006; Xu *et al.* 2007), conical tilt (Lanzavecchia *et al.* 2005), and multiple-axis tomography (Messaoudi *et al.* 2006). While these techniques reduce the missing wedge (to a missing pyramid, a missing cone, or a smaller missing region in general depending on how many tiltseries around different axes are combined), complete coverage is still not achieved. Furthermore, dose fractionation becomes increasingly problematic when two or more tiltseries are collected from the same imaging area, compromising the ability to align the images in the tiltseries accurately due to an exceedingly low SNR in individual images. In a recent proof-of-concept study, FIB milling was used to shape cellular material into a needle that could be fully rotated (*i.e.*, from −90° to 90°) (Narayan *et al.* 2012) and imaged by scanning electron microscopy (SEM). This study demonstrated that atom probe tomography (APT) (Miller *et al.* 2012) can be applied to chemically map freeze-dried cells with a thin metal coat in 3D. However, this was a unique experiment on unique equipment; it remains to be demonstrated whether an analogous approach could become widely applicable to frozen-hydrated cells using cryoET. In this direction, a recent study (Saghi *et al.* 2016) combined needle-shape FIB milling of the specimen with SEM imaging and tomographic reconstruction using CS. While these are exciting advances, added complications in data collection, storage, and processing preclude multiple-axis cryoET and APT of biological specimens from being routinely applied. A more promising proof of concept visualized bacterial cells by cryoET using a novel cylindrical holder (Palmer and Löwe 2014).

### Missing wedge compensation for subtomogram classification and alignment

**Fig. 7:**
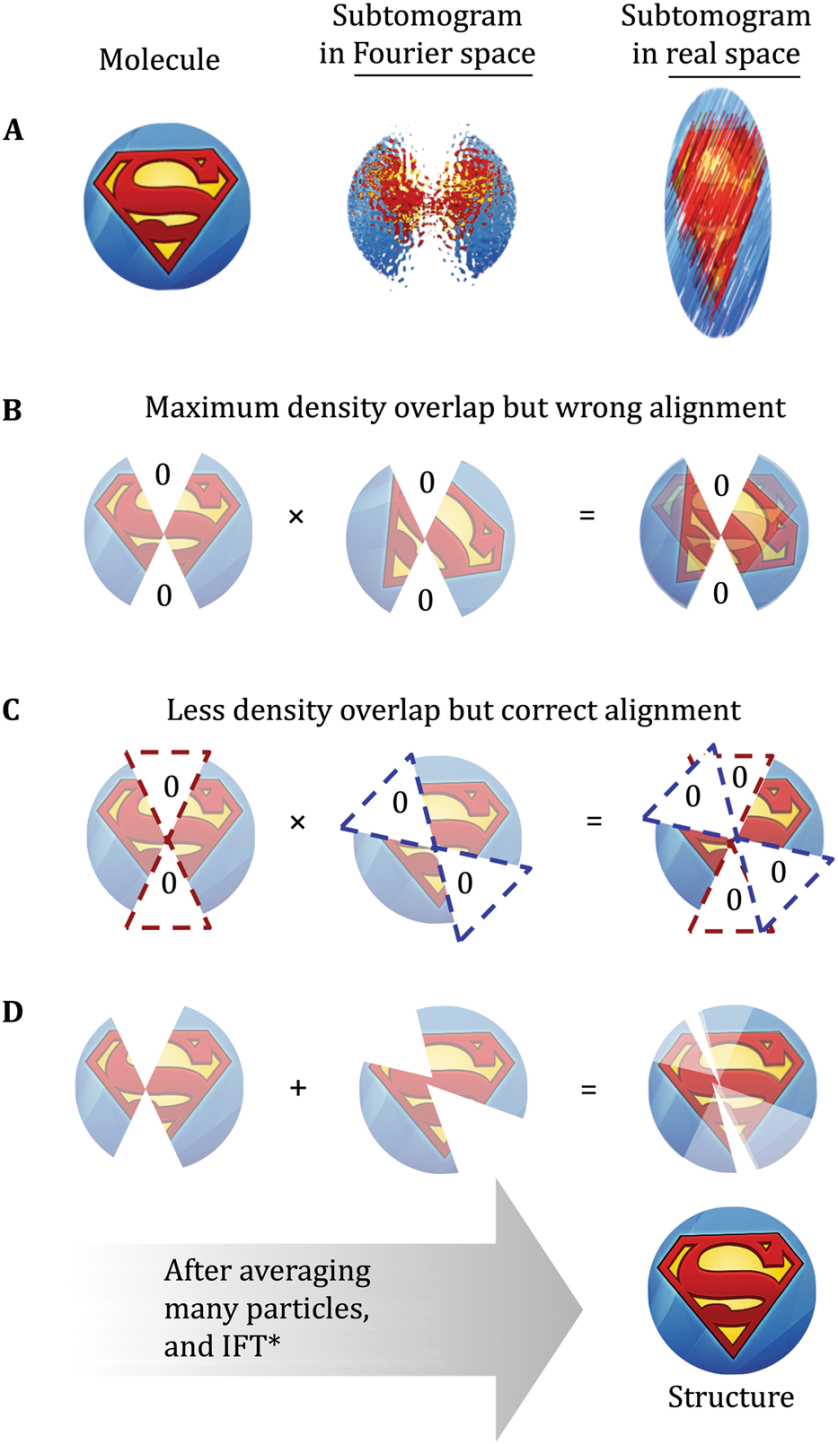
Missing wedge bias in Fourier space. **A** Cartoon representation of a molecule to image (*left*), its representation as a subtomogram in Fourier space after limited-angle tomographic reconstruction (*center*), and the effects that the “missing wedge” of information due to limited-angle imaging and dose fractionation due to limited tolerable electron dose have on the subtomogram in real space (*i.e.*, individual subtomograms suffer from elongation and high levels of noise). **B**, **C** Since the comparison metric most commonly used in SPT is cross correlation, greater density overlap tends to increase the similarity score during alignment. This “missing wedge bias” towards favoring larger density overlaps can cause misalignment of subtomograms; however, it can be compensated for by cross correlation map normalization or other methods. **D** After averaging many correctly aligned subtomograms in different orientations, the missing wedge can be “filled in”, and the associated elongation artifacts disappear while improving the SNR and the resolution of the macromolecular complex of interest. *Note* *:* The Fourier transform of the Superman logo would appear visually as an abstract and somewhat random pattern, in which it would be difficult to discern correct feature overlaps. For this reason, we have represented the image in Fourier space using the real-space image as a proxy to facilitate interpretation of the impact of missing wedge bias

In cryoSPT, subtomograms need to be correctly aligned to each other or to a common reference before they can be averaged coherently. Preventing “missing wedge bias” is an essential step to accomplish this. Without correction, the missing wedge is the strongest feature in individual subtomograms and tends to bias the alignment of any two given subvolumes, favoring orientations with maximum density overlap as opposed to optimizing the overlap of matching structural features (Fig. 7).

Given the current state of SPT, accurate classification of noisy subtomograms with a missing wedge remains one of the biggest challenges. The missing wedge, as well as missing information between tilts, can make accurate classification statistically impossible in specific cases. Popular techniques used in cryoEM SPA such as multivariate statistical analysis (MSA) (Van Heel and Frank 1981; Frank *et al.* 1982; Van Heel 1984) can be tricky to apply to SPT, because the missing wedge, which is often the strongest feature, is difficult to exclude when the particles have already been rotated for alignment. A classification technique for subtomograms based on 2D reprojections has been proposed to overcome the uncertainties in classification introduced by the missing wedge (Yu *et al.* 2010, 2013). While this method would still be, in principle, subject to the degeneracy problem that arises in SPA cryoEM (Fig. 1), it may allow for fast, initial 2D classification of particles from cellular tomograms without the concern of overlapping densities.

Multiple methods have been proposed to identify and compensate for the missing wedge. Those based on normalization of cross correlation maps are the simplest and have been used successfully for template matching (Frangakis *et al.* 2002) and SPT (Nicastro *et al.* 2006; Schmid *et al.* 2006). These methods have the advantage of not requiring explicit identification of “missing voxels” in Fourier space. Other algorithms identify the missing wedge by presumed *a priori* knowledge of its exact location (Stölken *et al.* 2011) or by selection of a threshold value in Fourier space to constrain correlation (Bartesaghi *et al.* 2008; Förster *et al.* 2008; Schmid and Booth 2008). The rationale behind thresholding is that, for individual subtomograms, ~95% of the structural information is concentrated in approximately the top 1% strongest Fourier coefficients (Amat *et al.* 2010). Other proposed methods use an estimate of the true structure (Heumann *et al.* 2011) or fill in the missing information with data derived from the population average (Bostina *et al.* 2007). However, the latter methods assume structural homogeneity among particles. On the other hand, methods relying on thresholding assume that Fourier voxels corresponding to the missing wedge have small values and thus a threshold can be easily assigned to identify them. One caveat inherent to the thresholding method is that simple operations such as filtration and masking can alter the values of voxels inside or outside the missing wedge in different ways, leading to misidentification of missing wedge voxels and therefore increasing subtomogram alignment error. A dynamic, resolution-dependent, per-volume thresholding method was recently introduced (Galaz-Montoya *et al.* 2016a), which produces much more robust identification of the missing wedge than the use of a single threshold value, and better alignment results than adaptive low-pass filtering using estimates of the resolution (Hrabe *et al.* 2012) or FSC curves directly (Galaz-Montoya *et al.* 2015).

## IMPROVING SINGLE PARTICLE TOMOGRAPHY

Measuring the defocus directly from experimental images, coupled with several other methodological improvements such as an optimized tilt-image acquisition scheme (Hagen *et al.* 2017) and damage compensation (Bammes and Jin 2014), also known as exposure filtering (Grant and Grigorieff 2015), as well as the usage of cutting-edge technology, are some of the latest improvements that have permitted cryoSPT to achieve ~4 Å resolution in the best case reported to date (Schur *et al.* 2016).

Ideally, one would like to produce a well-resolved 3D map with isotropic resolution for any specimen, be it particles extracted from cells, or macromolecules in solution. Covering the full 180° rotation about any single axis would yield a tiltseries without a missing wedge, but while this has been successfully implemented in material sciences, it is still not generally possible for biological specimens. Proof-of-concept methodologies that solve this problem have been proposed, such as combining FIB and SEM (Narayan *et al.* 2012; Saghi *et al.* 2016), or using a novel cylindrical specimen holder for cryoET (Palmer and Löwe 2014). Continued development of cylindrical holders or applying needle shaping of bulk specimens by FIB to cryoET could have a significant positive impact on cryoSPT, particularly for macromolecules *in situ*.

The ongoing development of tomographic reconstruction methods will also propel biological discoveries by cellular cryoSPT in the years to come. A current limiting factor is the reliable, automated identification of heterogeneous macromolecular complexes in crowded environments. Due to the missing wedge and the low SNR of cellular tomograms, automated particle picking is prone to bias and high false-positive rates (Yu and Frangakis 2014; Hrabe 2015; Kunz *et al.* 2015), though the use of neural networks has been proposed to reduce such issues (Yu and Frangakis 2011). Reliable automation of tiltseries reconstruction into tomograms will also increase the throughput of studies by cryoET (Mastronarde and Held 2017). Automated segmentation of features in tomograms remains poor and can be extremely subjective and time consuming when performed manually (Hecksel *et al.* 2016), particularly for thick frozen-hydrated specimens. The use of compressed sensing and other methodologies that improve the quality of tomographic reconstructions should facilitate more accurate particle picking and more objective, automated annotation of cellular tomograms.

Ongoing methodological developments and automation of algorithms on various fronts will also continue to improve image processing. For example, movie-mode data collection with relatively high contrast using DDDs now allows decoupling the decay in image quality due to radiation damage versus that stemming from beam-induced motions. Taking advantage of this, an “exposure filtering” method was recently proposed (Grant and Grigorieff 2015), which optimizes the SNR of every frame collected, thereby minimizing the deleterious effects of radiation damage. This idea was also proposed in an earlier study that filtered DDD frames based on cumulative exposure to compensate for radiation damage (Bammes and Jin 2014; Wang *et al.* 2014).

Since many subvolumes in random orientations are typically combined in SPT, there is seldom a need to use traditional small tilt steps of 1°–2° during data collection. As long as there are sufficient images to permit accurate alignment of the tiltseries, much larger tilt steps may be used in most cases (perhaps as high as 10°), depending on the size of the specimen. This can dramatically reduce data collection and processing times as well as data storage requirements, with no negative impact on final averages if enough subtomograms are averaged. Using a larger tilt step for cryoSPT also has the positive effect that each tilt image has a higher dose, permitting the application of some methods that require high contrast in each image of a tiltseries (Iwasaki *et al.* 2005; Bartesaghi *et al.* 2012; Zhang and Ren 2012). Unfortunately, to this day many cryoSPT studies continue to collect data using canonical parameters originally designed for cryoET of unique, non-repeating structures such as cells.

Several unconventional proposed improvements to general cryoEM have been reviewed in (Massover 2011) and might yield beneficial effects for cryoET and cryoSPT as well. For example, the development of specimen holders that are more stable and allow collecting data at slower exposure rates with “resting” periods in between exposures might make biological specimens more impervious to radiation damage (Chen *et al.* 2008; Karuppasamy *et al.* 2011).

Novel specimen grids (Rhinow and Kühlbrandt 2008; Pantelic *et al.* 2010; Yoshioka *et al.* 2010; Russo and Passmore 2016) made with materials that more readily dissipate charge and heat during imaging can minimize beam-induced specimen motions as well as the bubbling and distortions caused by radiation damage. The widespread adoption of such grids will also improve cryoET and cryoSPT and accelerate discoveries by these methodologies.

The problem of low-contrast at tolerable doses in cryoEM micrographs was first partially alleviated by the introduction of better illumination sources (field emission guns, opposed to metal filaments) (Tonomura *et al.* 1979) and the usage of large defocus settings for contrast enhancement (Erickson and Klug 1970). More recently, energy filters (Zhu *et al.* 1997) and phase plates (Danev and Nagayama 2001; Majorovits *et al.* 2007) have provided dramatic improvements in image contrast (Dai *et al.* 2013). Most significantly, the Volta phase plate (Danev *et al.* 2014) was recently used to demonstrate cryoSPT at subnanometer resolution (Khoshouei *et al.* 2016), averaging merely ~1400 ribosome subtomograms. Earlier phase plates caused strong ringing or “fringe” artifacts due to the cut-on frequency determined by the size of the central hole responsible for the phase shift, necessitating cumbersome computational correction (Kishchenko *et al.* 2015). Hole-less phase plates, such as the Volta phase plate, reduce such artifacts and are thus poised to revolutionize cryoET and cryoSPT. Unfortunately, a number of technical problems still remain that impede routine, straightforward use of this technology. The progressive change of phase with dose on Volta phase plates as well as the defocus gradient at high tilt both necessitate taking phase plates significantly out of focus. These factors greatly complicate CTF correction, particularly at high tilt (Sharp *et al.* 2017).

DDDs are revolutionizing the field and also provide images with better contrast compared to CCDs by virtue of their improved DQE, allowing for the correction of beam-induced blurring at the whole-frame, or at the per-particle level in SPA cryoEM. A step further, a local, anisotropic motion correction method recently proposed for SPA cryoEM (Zheng *et al.* 2016) might find applications in effectively unblurring lower-SNR images of highly tilted, non-repeating specimens, which sometimes cannot be corrected with current methods (Fig. 6).

Lastly, the application of hybrid techniques such as combining FIB milling (Wang *et al.* 2012; Villa *et al.* 2013; Mahamid *et al.* 2016) and correlative light microscopy (Zhang 2013; Hampton *et al.* 2017) with cryoET and cryoSPT has opened the window for observing dynamic cellular processes in regions of thick mammalian cells at nanometer resolution. Further developments to make these hybrid methodologies accessible and routine are likely to lead the next generation of insights into the biochemistry occurring within cells.

## Abbreviations

CryoET: Cryo-electron tomography
CTF: Contrast transfer function
DDD: Direct detection device
SPT: Single particle tomography
STA: Subtomogram averaging
